# Targeting SNARE-Mediated Vesicle Transport to Block Invadopodium-Based Cancer Cell Invasion

**DOI:** 10.3389/fonc.2021.679955

**Published:** 2021-05-21

**Authors:** Genya Gorshtein, Olivia Grafinger, Marc G. Coppolino

**Affiliations:** ^1^ Department of Molecular and Cellular Biology, University of Guelph, Guelph, ON, Canada; ^2^ Department of Biological Sciences, Sunnybrook Research Institute, Toronto, ON, Canada

**Keywords:** invadopodia formation, vesicle traffic, SNARE, membrane type 1–matrix metalloproteinase, invasion

## Abstract

During metastasis, cancer cells can invade extracellular matrix (ECM) through a process mediated by matrix-degrading protrusions of the plasma membrane, termed invadopodia. Formation of invadopodia correlates with cells’ invasive and metastatic potential, and thus presents a potential target for therapeutic approaches to target metastatic progression. Invadopodia formation is dependent on the recruitment of proteins involved in intracellular signaling, actin cytoskeleton remodeling, and proteolytic matrix modification. The latter includes matrix degrading enzymes such as MT1-MMP, MMP2, and MMP9. These essential invadopodium-associated enzymes are required for localized matrix degradation, and their localization at invadopodia is central to invadopodium-based cancer cell invasion. Soluble *N*-ethylmaleimide-sensitive factor attachment protein receptors (SNAREs) facilitate intracellular vesicle traffic, including that involved in the transport of invadopodium-associated proteins, and in so doing promote modification of ECM and modulation of signaling pathways involved in the movement of cancer cells. Specific SNARE complexes have been found to support invadopodia formation, and these complexes are, in turn, regulated by associated proteins that interact specifically with SNAREs. Targeting SNARE regulatory proteins thus provides a possible approach to disrupt SNARE-dependent delivery of invadopodial proteins, including MT1-MMP, to sites of ECM modification. Here, we review recent studies of SNARE regulators that hold potential as targets for the development of anti-metastatic therapies for patients burdened with invadopodia-forming cancer types.

## Invadopodia and Metastatic Progression

Metastatic progression is one of the most clinically challenging aspects of cancer, ultimately contributing to significant mortality (1). Much of the development of anti-cancer therapeutics has focused on anti-proliferative drugs to attenuate or shrink tumor growth. Treatment options that function to target the invasion and metastasis of cancer cells are extremely limited, however, and warrant further investigation (2). A better understanding of the cellular and molecular mechanisms involved in cancer cell invasion is necessary to advance the development of anti-invasion drugs, as agents to mitigate metastatic progression of cancer and increase survival of patients.

Invadopodia are sub-cellular, membrane-associated structures that mediate cancer cell invasion and facilitate metastasis. These cancer-specific protrusions function to degrade the extracellular matrix (ECM), allowing cancerous cells to invade through these barriers, breach tissue compartments, intravasate into blood and lymphatic systems, extravasate and subsequently colonize secondary tissue sites (3–5). The primary tumors of many metastatic cancers display increased expression of key proteins involved in invadopodia formation (e.g. Tks5, EGFR), compared to non-metastatic cancers (6–8). Furthermore, the invasive and metastatic potential of primary tumors often correlates with their ability to form invadopodia (9). Invadopodia thus represent attractive targets for the inhibition of cancer cell invasion as part of the metastatic cascade.

Invadopodia contain an F-actin core, which is enriched in actin modeling proteins such as Arp2/3, N-WASP, Tks5, and cortactin. Formation of invadopodia is also dependent on the localization and activation of epidermal growth factor receptor (EGFR) and integrins, which elicit intracellular signaling cascades that recruit and activate signaling molecules such as FAK and Src kinase (10, 11). Together, these proteins make up the core structure of invadopodia, integrate signaling pathways to induce localized actin polymerization within invadopodia pre-cursors, and support the formation of membrane protrusions. The microtubule network is also crucial to invadopodia formation and maturation (12, 13). Microtubules have been shown to have important roles in the regulation of MT1-MMP activity at invadopodia (14). As well, microtubules and microtubule-regulating proteins have been implicated in the modulation of focal adhesion dynamics and cell-ECM interaction at invadopodia (15, 16). Maturation of invadopodia corresponds with the delivery of matrix metalloproteinases MT1-MMP, MMP-2, and MMP-9, which gives these distinct structures their degradative phenotype by initiating ECM remodeling (17, 18). The delivery and localization of all invadopodia-associated proteins is essential for invadopodia formation and function, and is mediated by soluble N-ethylmaleimide-sensitive factor attachment protein receptors (SNAREs).

## SNARE-Mediated Vesicle Traffic and Invadopodium Formation

**Figure 1:**
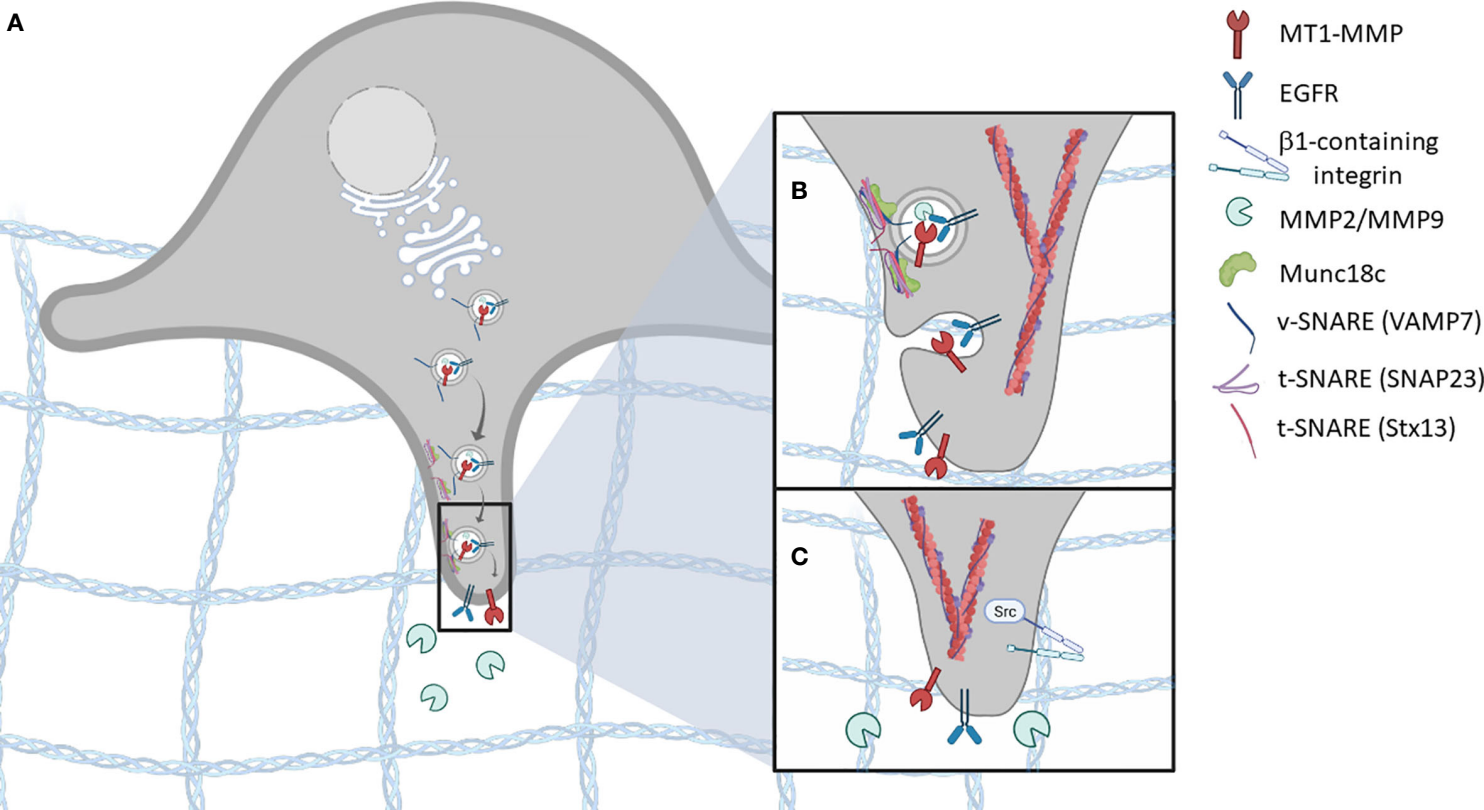
SNARE-mediated vesicle trafficking of invadopodium-associated proteins is essential for invadopodia formation. **(A)** vesicles containing invadopodial proteins, such as MT1-MMP, EGFR, and MMP2/MMP9, are trafficked to invadopodia pre-cursor sites. **(B)** As the vesicle comes in proximity to the target membrane, a SNARE complex is formed, involving t-SNAREs (Stx13, SNAP23) and v-SNAREs (VAMP7), that facilitates membrane fusion. **(C)** Membrane fusion results in the localization of invadopodium-associated proteins, at sites of ECM invasion. Additional invadopodial proteins are trafficked in a similar manner, including β1- containing integrins, which mediate downstream signaling pathways and promote invadopodia-based functions.

SNAREs are mediators of vesicle-based trafficking in cells and are central to both constitutive and regulated trafficking pathways. SNAREs form complexes between vesicle and target membranes, leading to fusion of the membranes and allowing delivery of vesicle contents to target compartment. In this manner, SNAREs contribute to the biosynthetic secretary pathway, endocytic recycling pathways, and regulated membrane traffic such as neurosecretion or insulin release. While much is known about SNARE-mediated membrane trafficking in some systems, our understanding of the membrane traffic in the context of cancer cells has significantly increased in recent years. SNARE-mediated trafficking of invadopodial proteins, to and from the plasma membrane, contributes to the remodeling of membranes and the localized enrichment of signaling components, adhesion receptors, and ECM degrading enzymes at sites where invadopodia form (**Figure 1**). Several studies point toward the role of specific SNAREs in trafficking invadopodium-associated proteins to promote cellular invasion and migration of malignant cancer cells (19–22).

The localization and activation of EGFR and β1 integrin to sites of cell-ECM attachment are important for invadopodia formation and function. Membrane trafficking pathways, involving SNAREs SNAP23 and Syntaxin13, have been shown to contribute to invadopodium formation through the delivery of EGFR and β1 integrin to the cell membrane (21). This trafficking pathway also delivers Src kinase, in association with EGFR and β1 integrin, to these sites (21). β1 integrin signaling stimulates SNARE complex formation, involving SNAP23 and syntaxin13, promoting the association of Src with EGFR, leading to receptor phosphorylation and activation (21). The association of Src, EGFR, and β1 integrin downstream of β1 integrin activation then promotes invadopodia formation and cellular invasion (23). Expression of SNAP23 constructs with cytoplasmic deletions and syntaxin13 dominant-negative mutants were shown to perturb invadopodia formation and cell invasion of ECM *in vitro* (21).

Secretion of MMPs correlates with the metastatic potential of cancers (6, 17, 18), and evidence suggests that MT1-MMP is a key protease that drives cancer cell invasion. It is clear that vesicle-mediated delivery of MT1-MMP promotes invadopodia maturation, and this is important for ECM remodeling and cell invasion (20, 22). For example, using several different cell culture models, SNARE complexes containing SNAP23, VAMP3 and syntaxin13 (20), or SNAP23, syntaxin4, and VAMP7, have been shown to contribute to invadopodia formation, by mediating the trafficking of MT1-MMP (22). These studies have demonstrated that both expression and function of the SNAREs were required for invadopodia formation and MT1-MMP localization to invadopodia (22). VAMP3 and syntaxin13 were also found to be involved in the secretion of MMP2 and MMP9 during ECM remodeling in invasive cancer cells (20). Collectively, these studies reveal that several, possibly overlapping, SNARE-mediated membrane trafficking pathways contribute to invadopodium formation and function, and how these pathways are interconnected and coordinated is an area of active investigation.

## Regulation of SNARE Function as a Therapeutic Target

The regulated assembly of SNARE complexes is necessary for the delivery of invadopodial proteins to the surface of cancer cells during cell invasion. Therapeutic targeting of SNARE proteins is therefore a potential approach for the inhibition of invadopodia-based invasion and subsequent metastatic spread of malignant cells; however, the diverse roles that SNARE proteins play in crucial physiological functions suggests that targeting SNAREs themselves may lead to detrimental side effects. While targeting a potential anti-SNARE therapy specifically to cancer cells is a theoretical possibility, a more attractive strategy might be to target regulators of SNARE function and complex formation to more specifically disrupt membrane trafficking pathways that are supporting invadopodium formation and invasive activity.

SNARE complex formation displays high specificity during membrane trafficking, providing fidelity in trafficking pathways (24), and this is achieved in part by post-translational regulation of SNARE activity. While much is known about the regulation of SNARE complex formation in some contexts, how SNAREs are regulated in invasive cancer cells is just emerging. One important mechanism for regulation of SNAREs is phosphorylation, and this has been well described in other systems (25, 26). In cancer cells, trafficking of MT1-MMP, involving SNAP23 and syntaxin4, has been shown to be modulated by phosphorylation of syntaxin4, with its dephosphorylation correlating with increased interaction with SNAP23 and increased invadopodium formation (22). The kinase(s) and phosphatase(s) responsible for phosphorylation and dephosphorylation of syntaxin4 in this context remain to be identified.

## Munc18c

SNAREs that have been implicated in cancer cell invasion have also been shown to be regulated by accessory proteins, including the Sec1/Munc18 (SM) family. SM family proteins are key regulators of SNARE-mediated membrane fusion, and they function by interacting with members of the syntaxin family of SNARE proteins (27). In one model, the binding of an SM protein to its cognate syntaxin is believed to modulate the syntaxin’s conformation to a primed open state (28). The “open” conformation of the syntaxin facilitates its association with other SNARE proteins necessary for the formation of a fusogenic SNARE complex. Munc18c is a known partner of syntaxin4 (29), a SNARE involved in the delivery of MT1-MMP- and EGFR-containing vesicles to invadopodial membranes (30). Munc18c has been reported to promote the formation of a syntaxin4-VAMP7-SNAP23 complex in MDA-MB-231 cells during invadopodia formation (22). A potential method to inhibit the delivery of invadopodial proteins to sites of invadopodia formation was studied, whereby Munc18c binding to endogenous syntaxin4 was perturbed. Exogenous expression of the 29 amino acid N-terminus of syntaxin4 (Stx4-N-term), containing the site that binds Munc18c, impaired the association of endogenous Munc18c and syntaxin4, possibly by competitively inhibiting syntaxin4-Munc18c binding (31). Cells expressing Stx4-N-term demonstrated decreased levels of syntaxin4-containing SNARE complexes, lower cell surface levels of MT1-MMP and EGFR, and inhibited invadopodium formation and matrix degradation.

## Gelsolin and Supervillin

Other Syntaxin4-regulatory molecules have been identified, including those in the gelsolin/villin superfamily (32). Gelsolin is a multifunctional actin-binding protein, which and can regulate the cytoskeleton by capping and severing F-actin filaments (33). Interestingly, gelsolin has also been found to play a role in the regulation of insulin exocytosis in pancreatic islet β-cells (34). Syntaxin4 mediates insulin granule docking at the plasma membrane of β-cells, forming a complex with SNAP25 and VAMP2 (35). Gelsolin was found to interact directly with the HA domain (amino acids 39-70) of syntaxin4 under resting conditions, suppressing SNARE complex formation (32). Upon glucose stimulation, gelsolin releases from Syntaxin4, allowing for the formation of cognate SNARE complexes necessary for insulin exocytosis. β cells overexpressing the HA domain of syntaxin4 were observed to secrete insulin in the absence of glucose, underscoring the importance of gelsolin in regulating insulin granule release.

Gelsolin and supervillin (another member of the gelsolin/villin superfamily) have been shown to localize to invadopodia where they regulate actin dynamics (36–38). Knockdown of both proteins in COS-7 and MDA-MB-231 cells was found to negatively affect MT1-MMP-dependent matrix degradation at invadopodia, as well as cellular invasion (37). Downregulation of gelsolin has also been shown to play a role in regulating the invasion and motility of MDA-MB-231 and PC-3 cells (39). Given gelsolin’s established role as a Syntaxin4-binding protein, it is plausible that members of the gelsolin/villin superfamily may be regulating SNARE complex formation to influence the delivery of cargo to the invadopodial membrane. Further research should be directed towards determining if gelsolin and supervillin associate with SNAREs during invadopodium-based cell invasion, and how perturbing their expression or function might influence invadopodial dynamics.

## Cdc42

The vesicle SNARE VAMP2 also has a potentially regulated role in invadopodia biogenesis. A complex of VAMP2-Syntaxin1A-SNAP25 plays a well understood role in insulin exocytosis in pancreatic β cells (40). Cdc42 can directly interact with VAMP2 in CHO-K1 cells, and this interaction promotes the formation of a complex with Syntaxin1A (40). Expression of a VAMP2 N-terminal peptide, corresponding to the binding site of cdc42, resulted in decreased insulin secretion in cells stimulated with glucose, demonstrating functional significance of VAMP2 regulation by cdc42. Additionally, VAMP2 has been shown to have a role in regulating cancer cell invasion, as knockdown of VAMP2 in HeLa cells resulted in decreased β1 integrin surface expression and cell migration (41). It is possible that cdc42 plays a role in regulating SNARE complex formation necessary for the delivery of β1 integrin-containing vesicles to the cell surface, possibly including sites of invadopodia formation.

## Concluding Remarks

Beyond Munc18c, gelsolin, supervillin, and cdc42, other SNARE regulatory proteins have yet to be described in the context of invadopodia biology. Several members of the SNARE family have been identified that mediate the fusion of vesicles containing invadopodial proteins (e.g. MT1-MMP, Src, EGFR) to the plasma membrane. VAMP2 (31, 41), VAMP3 (20, 42), VAMP7 (21, 22), SNAP23 (20, 31, 43), SNAP25 (43), syntaxin1 (43), syntaxin4 (22, 31), syntaxin6 (42), and syntaxin13 (21) are SNAREs whose expression has been shown to be upregulated in cancerous cells or have been identified to influence cellular invasion directly. Further investigation into proteins that associate with these SNAREs should be pursued, as these would represent potential druggable targets for impeding invadopodium-driven metastatic invasion.

**Figure 2:**
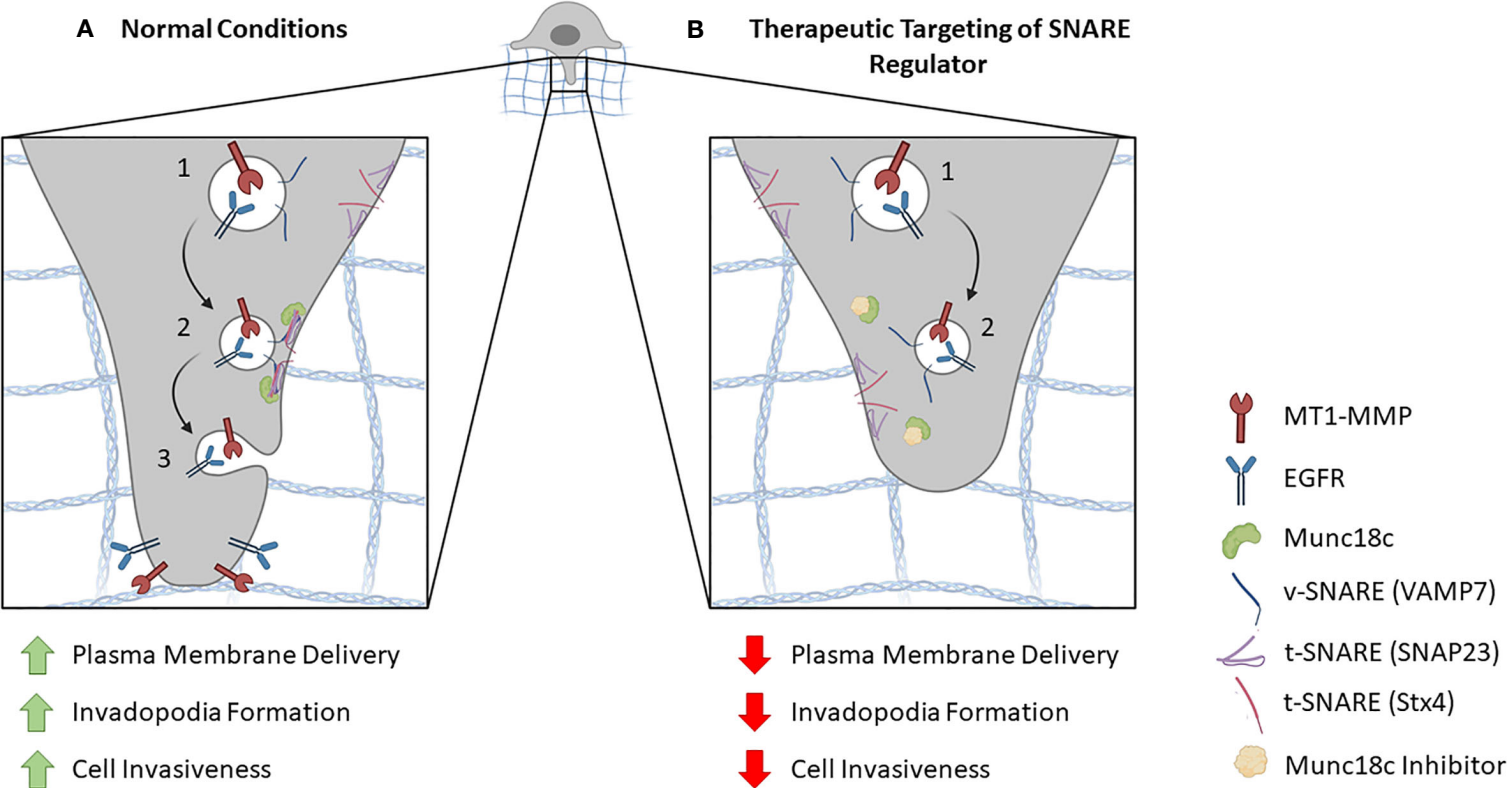
Proposed model for inhibiting SNARE-mediated membrane trafficking at invadopodia. **(A)** In normal conditions, components are delivered to invadopodia sites in a SNARE-regulated manner. (1) For example, vesicles containing MT1-MMP, EGFR and the *v*-SNARE Stx4 are depicted. (2) When the vesicle reaches its destination, a SNARE complex is formed by the *v-*SNARE and *t-*SNAREs (e.g. SNAP23, VAMP7) on the target membrane. This SNARE interaction is facilitated by the SNARE regulatory protein, Munc18c, which primes Stx4 for association with its cognate SNARE partners. (3) SNARE-mediated fusion between the vesicle and the invadopodial membrane results in the delivery of proteins necessary for invadopodia formation and cellular invasion. **(B)** Potential therapeutic targeting of a SNARE regulatory protein inhibitor. (1) As in A, vesicles containing MT1-MMP, EGFR and the *v*-SNARE Stx4 are depicted. (2) The presence of a Munc18c inhibitor impairs Stx4 involvement in SNARE complex formation, which reduces membrane fusion and delivery of invadopodial proteins leading to decreased invadopodia formation and cell invasion.

SNARE-dependent trafficking of proteins to invadopodia holds potential as a point of therapeutic intervention in metastatic progression. An effective approach to interfere with SNARE-dependent invadopodium formation and function is to target SNARE interactions with regulatory proteins that have been shown to be involved in invadopodia function (**Figure 2**). Such an approach has already been successful *in vitro* in MDA-MB-231 cells (31). These results provide a promising avenue for the development of anti-metastatic agents targeting SNARE regulatory molecules. Specific interactions between SNAREs and Munc18c, gelsolin, supervillin, as well as other unidentified SNARE regulatory proteins, represent potential targets to combat metastasis in patients with invadopodia-forming cancer subtypes.

## Author Contributions

GG and OG drafted the manuscript and generated the figures. MC edited and revised the manuscript and the figure. All authors contributed to conceptualization. All authors contributed to the article and approved the submitted version.

## Funding

NSERC Discovery Grant 05199: funded GG’s stipend and laboratory work.

## Conflict of Interest

The authors declare that the research was conducted in the absence of any commercial or financial relationships that could be construed as a potential conflict of interest.
